# An updated study-level meta-analysis of randomised controlled trials on proning in ARDS and acute lung injury

**DOI:** 10.1186/cc9403

**Published:** 2011-01-06

**Authors:** Fekri Abroug, Lamia Ouanes-Besbes, Fahmi Dachraoui, Islem Ouanes, Laurent Brochard

**Affiliations:** 1 ICU CHU F. Bourguiba, 1st June 1955 Str, University of Monastir, Monastir 5000, Tunisia; 2 Réanimation Médicale, AP-HP, Groupe hospitalier Albert Chenevier - Henri Mondor, Avenue du Général, Créteil, France; 3 Université Paris 12, Faculté de Médecine, Créteil, France; 4 INSERM unit 955, Equipe 13, Créteil, France

## Abstract

**Introduction:**

In patients with acute lung injury (ALI) and/or acute respiratory distress syndrome (ARDS), recent randomised controlled trials (RCTs) showed a consistent trend of mortality reduction with prone ventilation. We updated a meta-analysis on this topic.

**Methods:**

RCTs that compared ventilation of adult patients with ALI/ARDS in prone versus supine position were included in this study-level meta-analysis. Analysis was made by a random-effects model. The effect size on intensive care unit (ICU) mortality was computed in the overall included studies and in two subgroups of studies: those that included all ALI or hypoxemic patients, and those that restricted inclusion to only ARDS patients. A relationship between studies' effect size and daily prone duration was sought with meta-regression. We also computed the effects of prone positioning on major adverse airway complications.

**Results:**

Seven RCTs (including 1,675 adult patients, of whom 862 were ventilated in the prone position) were included. The four most recent trials included only ARDS patients, and also applied the longest proning durations and used lung-protective ventilation. The effects of prone positioning differed according to the type of study. Overall, prone ventilation did not reduce ICU mortality (odds ratio = 0.91, 95% confidence interval = 0.75 to 1.2; *P *= 0.39), but it significantly reduced the ICU mortality in the four recent studies that enrolled only patients with ARDS (odds ratio = 0.71; 95% confidence interval = 0.5 to 0.99; *P *= 0.048; number needed to treat = 11). Meta-regression on all studies disclosed only a trend to explain effect variation by prone duration (*P *= 0.06). Prone positioning was not associated with a statistical increase in major airway complications.

**Conclusions:**

Long duration of ventilation in prone position significantly reduces ICU mortality when only ARDS patients are considered.

## Introduction

The use of prone positioning during acute respiratory distress syndrome (ARDS) ventilation has a robust scientific ground and was evaluated in numerous randomised controlled trials (RCTs). Despite significant and sustained increase of oxygenation, prone positioning had no impact on mortality [1-4]. Most of these studies were underpowered, however, and meta-analyses intended to overcome the effects of inadequate sample sizes in individual RCTs failed to uncover any robust trend toward improved overall survival using prone positioning [5-9]. Yet from the first RCT evaluating prone ventilation (Prone-Supine Study), Gattinoni and colleagues highlighted in a *post-hoc *analysis that prone positioning reduced the 10-day mortality of patients with the highest disease severity (Simplified Acute Physiology Score II ≥50) [1]. A similar message is conveyed by selected analysis of the most severe patients in study-level meta-analyses [7,8]. These findings were recently reinforced by the conclusions of the Prone-Supine II Study suggesting that the most severe ARDS patients (defined by PaO_2_/FiO_2 _ratio <100 mmHg) could derive beneficial effects from prone ventilation with reduced mortality [10]. Consequently, recent meta-analyses of individual patient data obtained either from all published RCTs or from the four largest published RCTs showed unquestionably that the subgroup of the most severe patients (those with PaO_2_/FiO_2 _ratio <100 mmHg) had a significant reduction in mortality with prone ventilation [11,12].

Meta-analysis of individual patient data helps to avoid ecological bias, allows sufficiently powered subgroup analysis, and even allows powerful and reliable evaluation of treatment effects across individuals [13]. This type of meta-analysis, however, does not solve all problems encountered in study-level meta-analyses. Indeed, the accuracy of individual patient data depends on the quality (conduct) and similarity (design) of primary studies, and heterogeneity might still be present if trials are not sufficiently similar or carry potential sources of bias [13]. Moreover, individual patient data meta-analysis has frequently been shown to disclose divergent results from those of study-level aggregate meta-analysis [13,14].

In a previous aggregate meta-analysis we emphasised the substantial clinical (rather than statistical) heterogeneity of primary studies, making it difficult to conduct a study-level meta-analysis evaluating prone ventilation [5]. This heterogeneity resulted merely from ecological bias, which is caused by confounding across trials [15]. Ecological bias usually arises from within-group variability in covariates that may influence the outcome. In the particular setting of early studies on prone ventilation, ecological bias consisted of variable prone duration, mixed severity of acute lung injury, variable time-lapse between lung injury onset and inclusion, and lack of standardisation of co-interventions such as the lack of protective lung ventilation. Early studies were also vulnerable to treatment contamination, by allowing for crossover from one trial arm to another.

Given the large sample sizes of the initial studies, the heterogeneity in terms of severity as well as patient management heavily impacted the study-level meta-analyses [5-9]. Of note, the most recent RCTs - which learned from the shortcomings of early studies, and were able to incorporate recent knowledge advances regarding lung-protective ventilation - reached a consistent design that was sharply different from that of the large RCTs published earlier in the decade. Indeed, careful examination of these trials shows that they share the following common features: inclusion of the most severe patients (ARDS only, excluding acute lung injury (ALI) non-ARDS patients), and control for the most relevant confounders - that is, proning duration (usually >17 hours/day) and use of lung-protective ventilation [10,16,17]. Interestingly, each of these studies reported a substantial reduction in absolute risk of mortality varying between 9 and 15% but lacked power to reject a type II statistical error [10,16,17]. The possible minimisation of ecological bias therefore makes these new studies an interesting opportunity for a new updated study-level meta-analysis.

In the present article, we update our recent meta-analysis of the effects of prone positioning on intensive care unit (ICU) mortality. Along with a global meta-analysis, a subgroup meta-analysis is performed on the group of studies that restricted inclusion to only adult ARDS patients. We also explore the effects of proning duration.

## Materials and methods

### Search strategy and study selection

The search strategy and selection of studies are similar to those described in our previous meta-analysis [5]. Pertinent studies were independently searched in PubMed, EMBASE, CINAHL, and BioMedCentral (updated 30 March 2010), using the following MeSH and keyword terms: 'acute respiratory distress syndrome', 'acute lung injury', 'acute respiratory failure', and 'prone position ventilation'. RCTs that evaluated mechanical ventilation in prone versus supine positioning in adults with acute respiratory failure, ALI, or ARDS were included in the analysis. To minimise heterogeneity, we decided to keep only studies performed in adults. The rationale of proning in adults is in part based on homogenisation of the pleural pressure gradient and changes in chest wall compliance [18]. Whether this also occurs in children with a different chest wall configuration is not known. Studies conducted in infants were therefore not included.

### Data extraction and study characteristics

Three investigators (LO-B, FD and IO) independently evaluated studies for inclusion and abstracted data on methods and outcomes; disagreements were resolved by consensus between investigators. We extracted the study design, type of population and disease severity (assessed by the PaO_2_/FiO_2 _ratio), prone position duration on a 24-hour basis, and ICU mortality reported on an intention-to-treat basis. The methodological quality of each trial was evaluated using the five-point scale (0 = worst and 5 = best) as described by Jadad and colleagues [19]. Since all published meta-analyses have shown that prone ventilation was effective on oxygenation and prevention of ventilator-associated pneumonia, while the most recent one expressed doubts about its safety, we focused our analysis mainly on the effects of prone ventilation on both the ICU mortality and the procedure's complications.

### Statistical methods

ICU mortality was analysed by means of a random-effects model (assuming that the true effect could vary from trial to trial) to compute individual odds ratios (ORs) with 95% confidence intervals (CIs), and a pooled summary effect estimate was calculated. Since a clear change of primary study design has progressively occurred along with incorporation in everyday practice of new evidence generated by research, we evaluated the impact of publication date on the overall effect of prone ventilation by a cumulative meta-analysis. Indeed, this type of presentation roughly evaluates the trend over time for the overall effect of an intervention as new studies are published. We also compared the effect size of prone ventilation in two subgroups of studies: those that included all ALI patients, and those that included the most severe patients (ARDS patients). Noteworthy, this separation allows also comparison of earlier (before 2006) versus recent studies (after 2005), and studies that applied longer prone duration (≥17 hours/day) versus studies applying shorter prone duration.

Statistical interaction (heterogeneity effect) was sought by comparing the mean effect size for the two subgroups using the *z *test. Publication bias was assessed by visual inspection of the funnel plot and the Begg and Mazmudar rank correlation test. A relationship between study results (the effect size) and daily prone duration was sought with meta-regression. The incidence of complications related to prone positioning was also compared by means of a random-effects model. We analysed the incidence of major airways events corresponding to accidental extubation, and tracheal tube displacement with or without selective intubation. Statistical significance was set at the two-tailed 0.05 level for hypothesis testing and 0.10 for heterogeneity testing. Between-study heterogeneity was assessed using the *I*^2 ^measure. The meta-analysis was conducted using Comprehensive Meta Analysis v2 (Biostat, Eaglewood, NJ, USA). The present study was performed in compliance with the PRISMA guidelines (Additional file 1) and the review protocol has not been previously registered [20].

## Results

### Search results and trial characteristics

**Figure 1:**
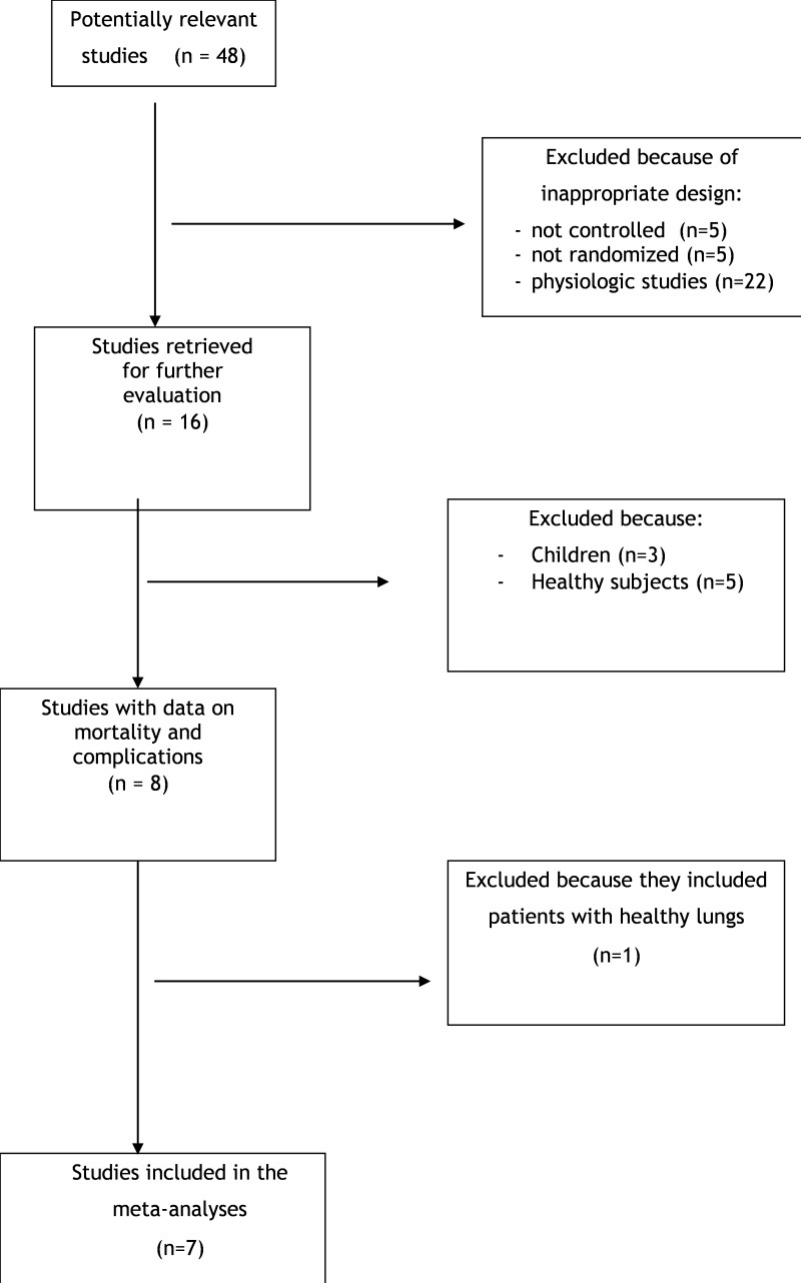
**Flow diagram of the meta-analysis**.

We identified 48 studies for detailed evaluation (Figure 1). Seven RCTs eventually met criteria for inclusion in the meta-analysis [1-3,10,16,17,21]. In comparison with our previous meta-analysis, one paediatric study was not included according to our new selection criteria [4], and three new RCTs issued during the past 2 years were added [10,17,21].

The study characteristics and methodological quality are presented in Table 1. These seven studies included 1,675 patients, of whom 862 were ventilated in the prone position for 7 to 24 hours/day. While early studies (published before 2006) included patients (*n *= 1,135) with a large spectrum of disease severity (ALI and ARDS), used a short duration of prone positioning (<17 hours), and did not use a protective lung ventilation, the four most recent trials were quite similar regarding patient severity (only ARDS patients were included, *n *= 540), applied the longest proning duration (17 to 24 hours/day), and ventilated patients with protective lung ventilation.

**Table 1:** Characteristics of the included studies

Trial	Disease	PaO_2_/FiO_2 _ratio	SAPS II	Population	Prone (*n*)	Supine (*n*)	Actual prone duration/day (hours)	Crossover allowed	Protective lung ventilation	Jadad score
Gattinoni_2001 [1]	ALI/ARDS (6%/94%)	127	40	304	152	152	7	Yes	No	3
Guerin_2004 [2]	ALI/ARDS (21%/31%) and other causes of ARF (pneumonia; acute on chronic ARF; CPE, coma)	153	46	791	413	378	8	Yes	No	3
Voggenreiter_2005 [3]	ALI/ARDS (45%/55%) (trauma)	222	NA	40	21	19	11	No	Yes	3
Mancebo_2006 [16]	ARDS	145	41	136	76	60	17	Yes	Yes	3
Chan_2007 [21]	ARDS	109	NA	22	11	11	24	No	Yes	1
Fernandez_2008 [17]	ARDS	120	38	40	21	19	20	Yes	Yes	3
Taccone_2009 [10]	ARDS	113	40	342	168	174	18	Yes	Yes	3
Total/mean		141 ± 39		1,675	862	813	15 ± 6			

ALI, acute lung injury; ARDS, acute respiratory distress syndrome; ARF, acute respiratory failure; CPE, cardiogenic pulmonary oedema; SAPS II, Simplified Acute Physiology Score II.

### Effects on mortality

**Figure 2:**
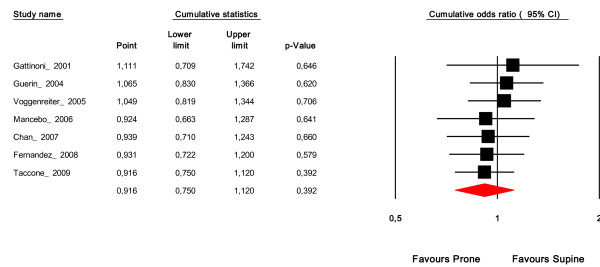
**Cumulative meta-analysis of prone ventilation on intensive care unit mortality**. The first row shows the effect based on one study, the second row shows the cumulative effects based on two studies, and so on. CI, confidence interval.

Pooling all studies was associated with a nonsignificant 9% reduction in ICU mortality (OR = 0.91, 95% CI = 0.75 to 1.1; *P *= 0.39; *I*^2 ^= 0%). Cumulative meta-analysis, which sorts studies chronologically, shows a progressive shift of the pooled summary effect of prone ventilation from a negative to a positive effect starting with the publication by Mancebo and colleagues, which was the first RCT to include ARDS patients only (Figure 2) [16].

**Figure 3:**
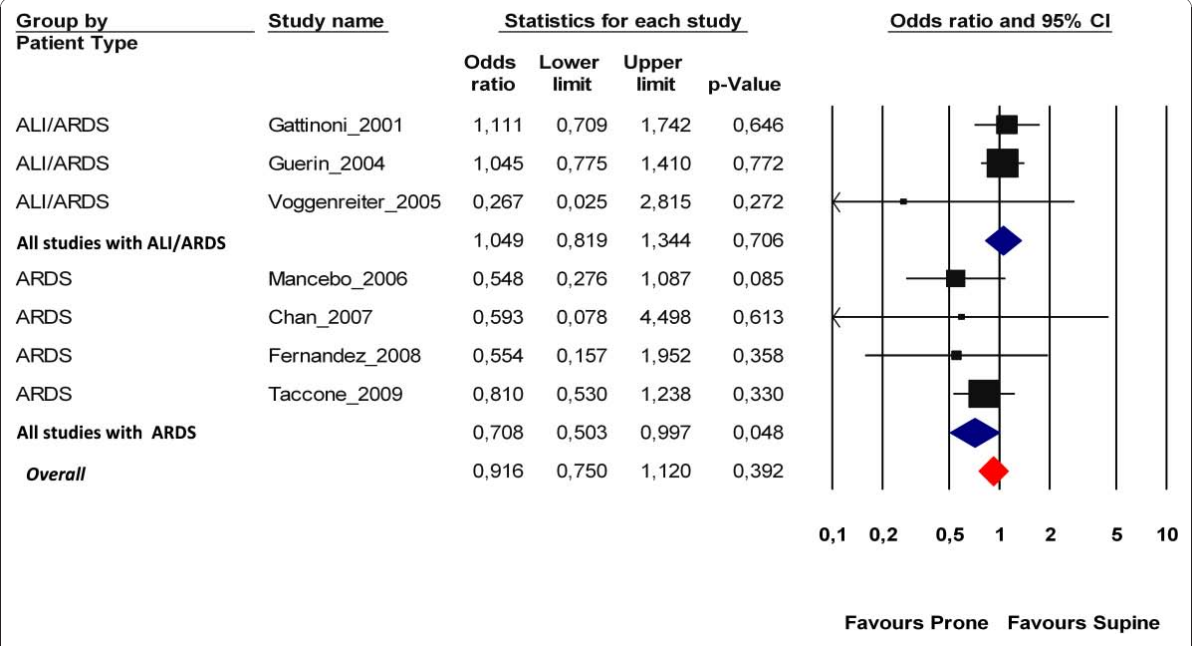
**Effects of prone ventilation on intensive care unit mortality**. Point estimates (by random-effects model) are reported separately for the groups of studies that included both acute lung injury (ALI) and acute respiratory distress syndrome patients (ARDS), those that included only ARDS patients, and the pooled overall effects of all meta-analysis-included patients. CI, confidence interval.

As anticipated, the effects of prone positioning were different in both subgroups considered according to disease severity (Figure 3). Proning had no significant effect in the earlier studies (three studies, *n *= 1,135 patients) that included patients with variable disease severity - that is, all ALI or hypoxemic patients (OR = 1.05; 95% CI = 0.82 to 1.34; *P *= 0.7; *I*^2 ^= 0%) - while it significantly reduced the ICU mortality rate in the four most recent studies (*n *= 540 patients) that included only patients with ARDS (OR = 0.71; 95% CI = 0.5 to 0.99; *P *= 0.048; number needed to treat = 11; *I*^2 ^= 0%). The *z *test of interaction was not significant (*z *= 1.87; *P *= 0.06), indicating that a heterogeneity of treatment effects between both subgroups was not certain. Funnel plot inspection did not suggest publication bias, and Begg's rank correlation test was not statistically significant (*P *= 0.23).

**Figure 4:**
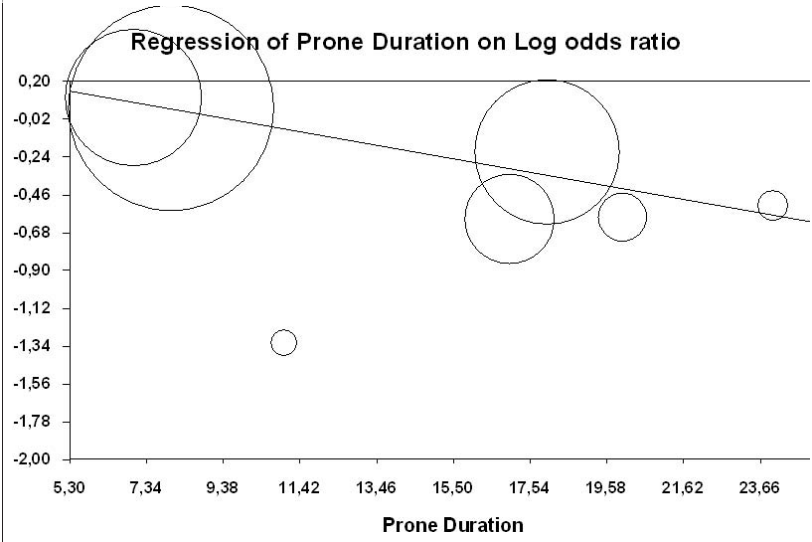
**Meta-regression analysis of effects of prone duration (actually applied in included studies) on mortality**. Log odds ratio plotted according to prone duration with the summary fixed-effects meta-regression (*z *= -1.88; *P *= 0.06). Each study is represented by a circle proportional to its weight in the meta-analysis reflecting the greatest impact on the slope of the regression line.

The result of a meta-regression that assessed the relationship between prone duration and effect size in included studies is presented in Figure 4. There was only a nonsignificant trend to explain effect size variation by actual prone duration (*z *= -1.88; *P *= 0.06).

### Adverse events

**Figure 5:**
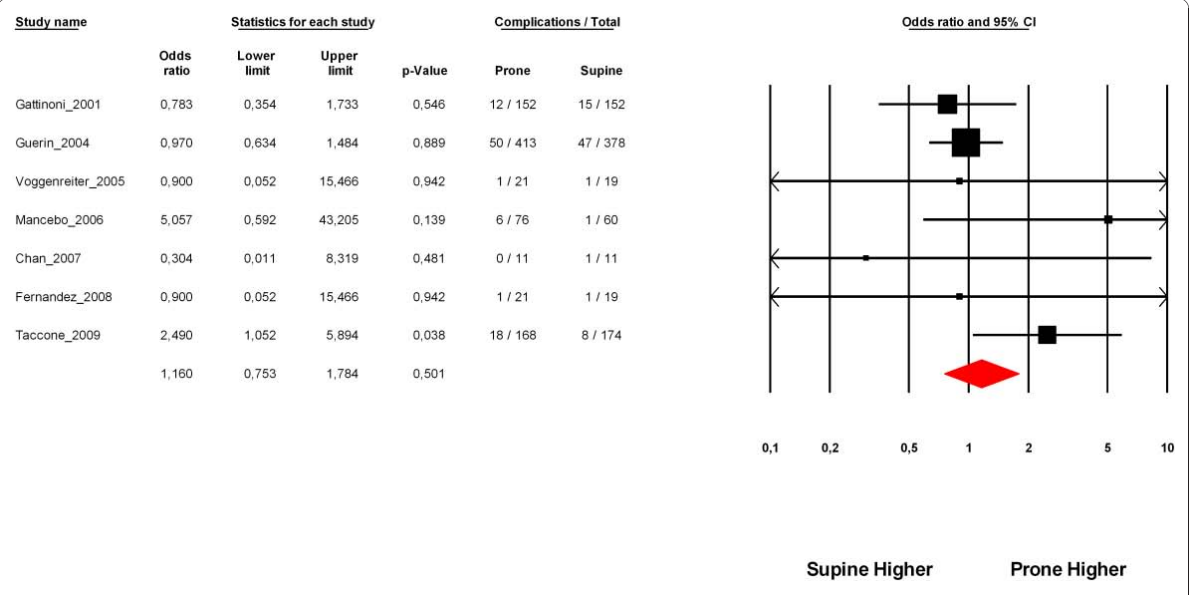
**Incidence of major airway complications**. CI, confidence interval.

All included RCTs reported data regarding airway complications related to prone positioning. The prone positioning was associated with a nonsignificant increase in the incidence of accidental extubation, selective intubation, or tracheal tube displacement (OR = 1.16; 95% CI = 0.75 to 1.78; *P *= 0.5) (Figure 5). The heterogeneity among trials was not significant (*I*^2 ^= 15%, *P *= 0.31).

## Discussion

The current meta-analysis shows that global analysis of RCTs assessing ventilation in the prone position in ALI/ARDS patients does not show a significant benefit on ICU mortality. The subgroup analysis stratified on the type of included patients in primary studies, however, disclosed a statistically significant reduction in mortality in the studies that restricted inclusion to only patients with ARDS, and not in those also enrolling patients with less disease severity. The comparison of the mean effect size between subgroups was close to significance (*P *= 0.06), however, which does not allow one to ensure that the effects of proning were significantly different between subgroups. Another confounder may also be the daily duration of ventilation in the prone position (*P *= 0.06). Prone positioning was not associated with an increase in major airway complications. The current study-level meta-analysis confirms and reinforces recent findings of individual patient data meta-analyses made by Sud and colleagues and Gattinoni and colleagues [11,12].

In many meta-analyses, the inclusion criteria are so broad that a certain amount of diversity among studies is inevitable. A study-level meta-analysis should anticipate this diversity and interpret the findings according to the results dispersion across the primary studies. We therefore applied the random-effects model, and computed a summary effect in subgroups of studies enrolling patients of variable lung injury severity, yielding important information on the peculiar effects of prone ventilation in the most severe patients.

A way to fully account for the ecological bias inherent to diversity of designs in primary studies is the performance of a meta-analysis using individual patient data [13]. Indeed, previous inferences on prone ventilation benefits for the most severe hypoxemic patients were recently confirmed by the meta-analyses from Sud and colleagues and from Gattinoni and colleagues showing reduced mortality rate in patients with PaO_2_/FiO_2 _ratio <100 mmHg [11,12]. This threshold was considered prospectively only in the study by Taccone and colleagues [10], however, while separation on this threshold basis was mostly retrospective for the other trials. Owing to increased risks of untoward effects, the authors recommended considering prone ventilation only in the most severe hypoxemia (despite a significant benefit up to PaO_2_/FiO_2 _ratio = 140 mmHg).

Our study used a different meta-analysis approach and stratified subgroups of studies according to the disease severity of included patients, rather than performing a subgroup analysis of included patients. This study reached the same conclusions as individual patient data meta-analyses, although our findings suggest that the benefits can go beyond the recommended threshold and concern all patients meeting ARDS criteria. A study-level meta-analysis like ours could therefore be an alternative for clinicians to detect true intervention effects (signals) despite differences among studies regarding participants, interventions, and co-interventions (noise) [22]. We should, however, recognise that such meta-analysis necessarily suffers some shortcomings - such as mixing in the same subgroup the early study by Gattinoni and colleagues [22], which included almost 93% ARDS patients, and that by Guerin and colleagues [22], which included only 30% of ARDS patients.

It is also difficult to control for important confounders such as the differences in prone duration, ventilation strategy, or associated treatments. Indeed, studies that included only ARDS patients also implemented lung-protective ventilation and longer prone duration, making it difficult to ascribe the observed reduction in ICU mortality to only one of these variables. Lung-protective ventilation has proved to lessen ventilation-induced lung injury and to reduce mortality, while longer prone duration helps to increase lung recruitment and enhances gas exchange [23,24]. Following Gattinoni and colleagues, however, we should admit that a strong physiological rationale underlies the fact that only the most severe forms of ALI (namely patients with ARDS) have physiological conditions for proning efficacy and might derive clinical benefit from prone ventilation [12]. Patients with ARDS indeed have a higher percentage of potentially recruitable lung, greater amounts of lung oedema, and a small portion of aerated lung [25]. Our working hypothesis prompting stratification of included studies according to the severity of acute lung injury (ARDS studies versus ALI/ARDS studies) therefore seems the most likely to account for the observed reduction in mortality in the ARDS subgroup.

The fact that the test of interaction yielded only a trend to different mean effect size of prone ventilation in the subgroup of ARDS patients when compared with studies that included all ALI is not surprising given that studies including ALI patients also enrolled a substantial proportion of patients with ARDS. Without specific studies enrolling only ALI non-ARDS patients, this type of effect comparison may be difficult. Apart from a type II statistical error, the nonsignificant test of interaction might also reflect a true lack of heterogeneity of prone ventilation effects. The use of confidence intervals is helpful to solve this uncertainty [26]. The 95% CI actually represents the range within which the true treatment effect falls 95% of the time. In the subgroup of studies enrolling only ARDS patients, the CI around the point estimate suggests that the reduction of mortality by prone ventilation could not be less than 1%. Similarly, the CI boundaries for the effect of prone ventilation in ALI/ARDS studies do not exclude a reduction by 18% in the mortality in such patients.

Our cumulative meta-analysis shows that beneficial effects of prone ventilation have progressively become apparent as new studies have been published. This finding suggests that the gradual incorporation of research advances (protective lung ventilation, inclusion of homogeneous groups of severity, standardisation of length of proning, and so forth) influenced the trend toward an apparent benefit from prone positioning. This cumulative meta-analysis also shows that the size effect of prone ventilation on mortality has become almost constant since 2006 following the study by Mancebo and colleagues [16]. Subsequent studies have merely contributed to improve precision of this effect as reflected by a progressive narrowing of the confidence interval. Increased precision rather than substantial alteration in size effect is probably what would be added by any new study on prone ventilation. Furthermore, such a study would be difficult to complete given inclusion barriers encountered by most of the recent RCTs. Meanwhile, the present aggregate meta-analysis and the recent individual patient data meta-analyses provide compelling evidence to recommend prone ventilation in ARDS patients.

Our meta-analysis did not disclose a statistically significant increase in major airway complications of prone positioning. The most recent RCT (Prone-Supine II Study), however - which should be regarded as the most reliable reflection of real-life practice - recorded a higher incidence of adverse events associated with prone positioning [10]. This concerned not only airway complications but also the need for increased sedation, transient desaturation or hypotension, and displacement of vascular lines. Accordingly, caution should be kept during the manoeuvre - which should be applied only in the most severe patients.

The survival difference between ALI/ARDS studies and only ARDS studies might have additional possible contributors, other than the disease severity. The ALI/ARDS studies are the older studies, characterised by several methodological differences such as the absence of relevant co-treatments (lung-protective mechanical ventilation strategy), other criteria of enrolment (time window between ARDS criteria and enrolment), and so forth. The main difference is that the length of the proning treatment - which may constitute an important determinant of the survival benefit - is profoundly different between older studies (shorter duration) and newer studies (longer duration). Indeed, alveolar recruitment in the prone position is a time-dependent phenomenon [23]. Our study therefore cannot ascertain whether the enrolment criteria by themselves explain the results, and suggests that proning duration also played a role. We addressed the practical issue of the optimal proning duration by a meta-regression analysis. We found only a trend towards an interaction between longer proning duration and reduction in mortality. The initial studies by Guerin and colleagues [2] and Gattinoni and colleagues [1] had the greatest impact on the slope of the regression line. The subgroup of studies including only ARDS patients also applied the longest proning durations (17 to 24 hours/day). Hence, although proning duration seems to play a role in the outcome effect, the present analysis cannot definitely confirm this effect.

## Conclusions

The present study-level meta-analysis based on an observation (each of the most recent RCTs reported a substantial, although nonsignificant, reduction in ICU mortality by prone ventilation) and a working hypothesis (only ARDS patients would derive benefit from prone ventilation) tried to overcome primary trial heterogeneity by a subgroup meta-analysis of studies that restricted inclusion to only ARDS. This meta-analysis shows that prone ventilation significantly reduces ICU mortality in ARDS patients and suggests that long prone durations should be applied.

## Key messages

• The use of prone positioning during ARDS ventilation has a robust scientific ground.

• Available RCTs that were frequently underpowered failed to document an impact on mortality mainly because they included patients with a wide spectrum of disease (ALI and ARDS) and applied variable length of prone positioning.

• Study-level meta-analyses published so far only suggested beneficial effects on mortality.

• Meta-analyses of individual patient data have recently shown that prone positioning could reduce ICU mortality in the subgroup of the most severe patients (PaO_2_/FiO_2 _ratio <100 mmHg).

• Using a subgroup analysis focusing on trials that restricted inclusion to only ARDS patients, our study-level meta-analysis shows that prone positioning reduces ICU mortality in patients with ARDS.

## Abbreviations

ALI: acute lung injury; ARDS: acute respiratory distress syndrome; CI: confidence interval; FiO_2_: inspiratory fraction of oxygen; ICU: intensive care unit; PaO_2_: arterial partial pressure of oxygen; OR: odds ratio; RCT: randomised controlled trial.

## Competing interests

The authors declare that they have no competing interests.

## Authors' contributions

FA conducted the literature searches, selected studies, extracted data, assessed study quality, prepared initial and subsequent drafts of the manuscript, and integrated comments from other authors into revised versions of the manuscript. LO-B, FD, and IO screened abstracts, selected studies meeting inclusion criteria, extracted data, and assessed study quality. FA and LO-B carried out the statistical analyses with input from IO, FD and LB. LB provided methodological guidance on drafting the manuscript. All authors read and approved the final manuscript.

## Supplementary Material

Additional file 1 **PRISMA checklist**. Checklist according to the PRISMA guidelines. Click here for file

## References

[B1] Gattinoni L Tognoni G Pesenti A Taccone P Mascheroni D Labarta V Malacrida R Di Giulio P Fumagalli R Pelosi P Brazzi L Latini R Effect of prone positioning on the survival of patients with acute respiratory failure N Engl J Med 2001 345 568 573 10.1056/NEJMoa010043 11529210

[B2] Guerin C Gaillard S Lemasson S Ayzac L Girard R Beuret P Palmier B Le QV Sirodot M Rosselli S Cadiergue V Sainty JM Barbe P Combourieu E Debatty D Rouffineau J Ezingeard E Millet O Guelon D Rodriguez L Martin O Renault A Sibille JP Kaidomar M Effects of systematic prone positioning in hypoxemic acute respiratory failure: a randomized controlled trial JAMA 2004 292 2379 2387 10.1001/jama.292.19.2379 15547166

[B3] Voggenreiter G Aufmkolk M Stiletto RJ Baacke MG Waydhas C Ose C Bock E Gotzen L Obertacke U Nast-Kolb D Prone positioning improves oxygenation in post-traumatic lung injury - a prospective randomized trial J Trauma 2005 59 333 341 discussion 341-343 10.1097/01.ta.0000179952.95921.49 16294072

[B4] Curley MA Hibberd PL Fineman LD Wypij D Shih MC Thompson JE Grant MJ Barr FE Cvijanovich NZ Sorce L Luckett PM Matthay MA Arnold JH Effect of prone positioning on clinical outcomes in children with acute lung injury: a randomized controlled trial JAMA 2005 294 229 237 10.1001/jama.294.2.229 16014597 PMC1237036

[B5] Abroug F Ouanes-Besbes L Elatrous S Brochard L The effect of prone positioning in acute respiratory distress syndrome or acute lung injury: a meta-analysis. Areas of uncertainty and recommendations for research Intensive Care Med 2008 34 1002 1011 10.1007/s00134-008-1062-3 18350271

[B6] Tiruvoipati R Bangash M Manktelow B Peek GJ Efficacy of prone ventilation in adult patients with acute respiratory failure: a meta-analysis J Crit Care 2008 23 101 110 10.1016/j.jcrc.2007.09.003 18359427

[B7] Alsaghir AH Martin CM Effect of prone positioning in patients with acute respiratory distress syndrome: a meta-analysis Crit Care Med 2008 36 603 609 10.1097/01.CCM.0000299739.98236.05 18216609

[B8] Kopterides P Siempos II Armaganidis A Prone positioning in hypoxemic respiratory failure: meta-analysis of randomized controlled trials J Crit Care 2009 24 89 100 10.1016/j.jcrc.2007.12.014 19272544

[B9] Sud S Sud M Friedrich JO Adhikari NK Effect of mechanical ventilation in the prone position on clinical outcomes in patients with acute hypoxemic respiratory failure: a systematic review and meta-analysis CMAJ 2008 178 1153 1161 18427090 10.1503/cmaj.071802 PMC2292779

[B10] Taccone P Pesenti A Latini R Polli F Vagginelli F Mietto C Caspani L Raimondi F Bordone G Iapichino G Mancebo J Guerin C Ayzac L Blanch L Fumagalli R Tognoni G Gattinoni L Prone positioning in patients with moderate and severe acute respiratory distress syndrome: a randomized controlled trial JAMA 2009 302 1977 1984 10.1001/jama.2009.1614 19903918

[B11] Sud S Friedrich JO Taccone P Polli F Adhikari NK Latini R Pesenti A Guerin C Mancebo J Curley MA Fernandez R Chan MC Beuret P Voggenreiter G Sud M Tognoni G Gattinoni L Prone ventilation reduces mortality in patients with acute respiratory failure and severe hypoxemia: systematic review and meta-analysis Intensive Care Med 2010 36 585 599 10.1007/s00134-009-1748-1 20130832

[B12] Gattinoni L Carlesso E Taccone P Polli F Guerin C Mancebo J Prone positioning improves survival in severe ARDS: a pathophysiologic review and individual patient meta-analysis Minerva Anestesiol 2010 76 448 454 20473258

[B13] Reade MC Delaney A Bailey MJ Harrison DA Yealy DM Jones PG Rowan KM Bellomo R Angus DC Prospective meta-analysis using individual patient data in intensive care medicine Intensive Care Med 2010 36 11 21 10.1007/s00134-009-1650-x 19760395 PMC7079872

[B14] Riley RD Lambert PC Abo-Zaid G Meta-analysis of individual participant data: rationale, conduct, and reporting BMJ 2010 340 c221 10.1136/bmj.c221 20139215

[B15] Berlin JA Santanna J Schmid CH Szczech LA Feldman HI Individual patient- versus group-level data meta-regressions for the investigation of treatment effect modifiers: ecological bias rears its ugly head Stat Med 2002 21 371 387 10.1002/sim.1023 11813224

[B16] Mancebo J Fernandez R Blanch L Rialp G Gordo F Ferrer M Rodriguez F Garro P Ricart P Vallverdu I Gich I Castano J Saura P Dominguez G Bonet A Albert RK A multicenter trial of prolonged prone ventilation in severe acute respiratory distress syndrome Am J Respir Crit Care Med 2006 173 1233 1239 10.1164/rccm.200503-353OC 16556697

[B17] Fernandez R Trenchs X Klamburg J Castedo J Serrano JM Besso G Tirapu JP Santos A Mas A Parraga M Jubert P Frutos F Anon JM Garcia M Rodriguez F Yebenes JC Lopez MJ Prone positioning in acute respiratory distress syndrome: a multicenter randomized clinical trial Intensive Care Med 2008 34 1487 1491 10.1007/s00134-008-1119-3 18427774

[B18] Pelosi P Tubiolo D Mascheroni D Vicardi P Crotti S Valenza F Gattinoni L Effects of the prone position on respiratory mechanics and gas exchange during acute lung injury Am J Respir Crit Care Med 1998 157 387 393 9476848 10.1164/ajrccm.157.2.97-04023

[B19] Jadad AR Moore RA Carroll D Jenkinson C Reynolds DJ Gavaghan DJ McQuay HJ Assessing the quality of reports of randomized clinical trials: is blinding necessary? Control Clin Trials 1996 17 1 12 10.1016/0197-2456(95)00134-4 8721797

[B20] Moher D Liberati A Tetzlaff J Altman DG Preferred reporting items for systematic reviews and meta-analyses: the PRISMA statement Ann Intern Med 2009 151 264 269 W264 19622511 10.7326/0003-4819-151-4-200908180-00135

[B21] Chan MC Hsu JY Liu HH Lee YL Pong SC Chang LY Kuo BI Wu CL Effects of prone position on inflammatory markers in patients with ARDS due to community-acquired pneumonia J Formos Med Assoc 2007 106 708 716 10.1016/S0929-6646(08)60032-7 17908660

[B22] Davidoff F Heterogeneity is not always noise: lessons from improvement JAMA 2009 302 2580 2586 10.1001/jama.2009.1845 20009058

[B23] Reutershan J Schmitt A Dietz K Unertl K Fretschner R Alveolar recruitment during prone position: time matters Clin Sci (Lond) 2006 110 655 663 10.1042/CS20050337 16451123

[B24] Ventilation with lower tidal volumes as compared with traditional tidal volumes for acute lung injury and the acute respiratory distress syndrome. The Acute Respiratory Distress Syndrome Network N Engl J Med 2000 342 1301 1308 10.1056/NEJM200005043421801 10793162

[B25] Gattinoni L Caironi P Cressoni M Chiumello D Ranieri VM Quintel M Russo S Patroniti N Cornejo R Bugedo G Lung recruitment in patients with the acute respiratory distress syndrome N Engl J Med 2006 354 1775 1786 10.1056/NEJMoa052052 16641394

[B26] Wyer PC Keitz S Hatala R Hayward R Barratt A Montori V Wooltorton E Guyatt G Tips for learning and teaching evidence-based medicine: introduction to the series CMAJ 2004 171 347 348 15313994 10.1503/cmaj.1031665 PMC509048

